# Current tobacco use and susceptibility to using tobacco among non-users of tobacco: A cross-sectional study among school-going adolescents in Sierra Leone

**DOI:** 10.18332/tid/157091

**Published:** 2023-01-30

**Authors:** Peter B. James, John A. Kabba, Abdulai J. Bah, Ayesha Idriss, Chenai Kitchen, Eugene B. Conteh, Michael Lahai, Philip A. Dalinjong

**Affiliations:** 1 National Centre for Naturopathic Medicine, Faculty of Health, Southern Cross University, Lismore, Australia; 2 Faculty of Pharmaceutical Sciences, College of Medicine and Allied Health Sciences, University of Sierra Leone, Freetown, Sierra Leone; 3 Department of Pharmacy Administration and Clinical Pharmacy, School of Pharmacy, Xi’an Jiaotong University, Xi’an, China; 4 Faculty of Basic Medical Sciences, College of Medicine and Allied Health Sciences, University of Sierra Leone, Freetown, Sierra Leone; 5 Institute for Global Health and Development, Queen Margaret University Edinburg, Musselburgh, United Kingdom; 6 Navrongo Health Research Centre, Navrongo, Ghana

**Keywords:** tobacco use, adolescents, Sierra Leone, low- and middle-income countries, susceptibility to using tobacco

## Abstract

**INTRODUCTION:**

Tobacco use is a global health threat associated with a high disease burden and death. Current tobacco use and susceptibility to using tobacco products among adolescents who are potential adult tobacco users have not been explored in Sierra Leone. Thus, we aimed to estimate the prevalence and correlates of current tobacco use and tobacco non-users susceptibility to using tobacco amongst high school students in Sierra Leone.

**METHODS:**

We used data obtained from the 2017 Sierra Leone Global Youth Tobacco Survey (GYTS), which presented information collected from 6680 students aged 11–17 years nationwide. Gender-based correlates of current use and susceptibility to using tobacco among non-tobacco users were determined by complex sample logistic regression analyses. Adjusted odds ratios (AOR) and respective 95% confidence intervals (CIs) are reported. A p<0.05 was considered significant.

**RESULTS:**

The prevalence of current tobacco use among high school adolescents in Sierra Leone was 24.6%, higher in males (27.9%) than in females (18.6%). Male (AOR=1.50; 95% CI: 1.18–1.91), parental smoking (AOR=1.73; 95% CI: 1.32–2.26) exposure to household secondhand smoke (AOR=1.82; 95% CI: 1.27–2.62), having peers who smoke (AOR=2.24; 95% CI: 1.51–3.31) were more likely to be currently using tobacco. The overall tobacco non-users susceptibility to using tobacco among adolescents in Sierra Leone was 18.2% (males 18.0%, females 18.5%). Exposure to tobacco promotion (AOR=1.50; 95% CI: 1.06–2.10) and non-exposure to anti-smoking education (AOR=1.39; 95% CI: 1.05–1.86) were significantly associated with tobacco non-users’ susceptibility to using tobacco.

**CONCLUSIONS:**

Our study suggests that one in four school-going adolescents currently uses tobacco, with nearly one in five non-users susceptible to using tobacco. Given the high prevalence of tobacco product use among adults in Sierra Leone, our findings highlight the need for policies and interventions to prevent tobacco use behavior among adolescents, aimed at averting tobacco use in adulthood.

## INTRODUCTION

Tobacco use is considered a global public health threat. Currently, there are 1.3 billion people using tobacco, and 80% of them reside in low- to middle-income countries, where tobacco-related morbidity and mortality are the highest, and this figure is expected to rise to 1.7 billion by 2025^1^. It is estimated that eight million people die every year from health conditions for which tobacco use is considered the major cause, and more than three-quarters of deaths are due to direct tobacco use^2^. Chronic tobacco use is a leading risk factor for many preventable chronic diseases in children and young adults with short-term (nicotine addiction, sudden infant death syndrome, respiratory and atopic conditions) and long-term (stroke, chronic respiratory diseases, and cancers) repercussions^1^. Aside from its health impact, a high economic cost is associated with tobacco use, and this includes the health cost linked with managing diseases due to tobacco use and the loss in productivity due to tobacco-related morbidity and mortality^2^.

In Sub-Saharan Africa, the prevalence of tobacco use is relatively low but increasing^3^. Such characteristics make Sub-Saharan Africa a potential market for multinational tobacco companies to sell their products. The increasing use of tobacco in the region is further exacerbated by globalization, increased youth population and high illiteracy levels in the region^4^. Sierra Leone is one country that shares similar characteristics. A study conducted by Samai et al.^5^ reported that 26% and 8% of adults use tobacco and smokeless tobacco products, respectively, in Sierra Leone. The same study reported that more than two-thirds of non-smokers were exposed to secondhand smoke at home or at their workplace. Another study conducted by Lisk et al.^6^ in Freetown and three villages in the northern province of Sierra Leone reported that the prevalence of tobacco smoke (cigarette) and smokeless tobacco use were 21.7% and 17.7%, respectively. A recent United Nations Development Program (UNDP) report estimated that more than 3300 deaths are linked to tobacco-related diseases every year and that a quarter of those deaths are among the poor. The same report estimated that tobacco use cost 1.5% of the Sierra Leone GDP in 2017, and this cost includes healthcare cost of SLL 108.4 billion (about US$ 5.64 million), lost productivity due to morbidity, mortality, and workplace smoking (SLL 295.5 billion). Such statistics create a challenge to public health, which is augmented by the fact that non-communicable diseases are on the rise^7^. Also, despite that a national non-communicable disease (NCD) policy and strategic plan was developed in 2013, the country’s capacity to prevent, control, and manage the growing NCD burden is limited^7^. This has been associated with limited funding from government and donor partners, structural and financial barriers for users, maldistribution, limited training of the health workforce, and limited access to quality-assured medicines^8^.

Sierra Leone is a signatory to the World Health Organization (WHO) Framework Convention on Tobacco Control (WHO FCTC). The WHO FCTC provides measures to protect the public from social, environmental, and economic consequences of tobacco use. These measures include reducing demand (taxation, bans on smoking in all public places, public education) and supply (illegal trade in tobacco products and sales to and by minors) of tobacco^9^. As of 2018, Sierra Leone has only imposed a 30% excise tax on tobacco products, below the 70% excise tax recommended by WHO^10^. Also, anti-tobacco media campaigns have been implemented, although they have not been aired on mass media outlets. Measures such as, mandating tobacco products to carry health warnings that cover 50% of the packaging products and ban on all forms of tobacco advertising, promotion, and sponsorship, have not yet been implemented^10^. A bill called The Tobacco and Nicotine Control Act of 2022 was enacted into law in August 2022^11^. This law aims at regulating the production, manufacturing, importation, packaging and labelling, advertising, promotion and sponsorship, and sale and use of tobacco, tobacco products and other nicotine products in Sierra Leone^11^. More importantly, this law bans the use of tobacco in public places^11^.

Along with strong tobacco control measures targeting tobacco users, it is critical to implement interventions targeting non-tobacco users susceptible to tobacco use or initiating tobacco use behavior. Previous research has indicated that susceptibility to using tobacco is strongly associated with experimenting with a tobacco product compared to family history of using tobacco products^12^, with youths being more likely to be established users^13^. A previous global study found that 1 in 8 never-smoking youths was susceptible to smoking, of which 7.2% and 5.3% were males and females, respectively^14^. Although a comprehensive study has been conducted in Africa on this subject^3^, none to our knowledge has so far been conducted in Sierra Leone.

Given that the burden of tobacco use in Sierra Leone is increasing^5,15,16^, and many young people start smoking during adolescence^17^, there is a need to investigate tobacco use behavior among adolescents to inform policy, public health interventions and advocacy. Currently, studies on tobacco use among adolescents are scarce. A study by Bangura et al.^18^ reported that the prevalence of cigarette use was 3.2% among school-going pupils in Bo, southern Sierra Leone^18^. However, this study has certain key limitations. Firstly, it was conducted more than two decades ago, and therefore, its findings may no longer be valid, given the demographic transitions and increased urbanization that have occurred in Sierra Leone over the years. Secondly, the study findings are not representative of the whole country, given that it was only conducted in Bo, the second city of Sierra Leone. Also, other nationwide studies have only explored tobacco use among the adult population^5,15^. Using data from the 2017 Sierra Leone Global Youth Tobacco Survey (GYTS), we estimated the prevalence and correlates of current tobacco use and neversmoking youth susceptibility to using tobacco among high school students in Sierra Leone.

## METHODS

### Study setting, design, and population

We obtained our data from the Sierra Leone GYTS conducted in 2017, which are publicly available at WHO - Global Tobacco Surveillance System (WHO - GTSS) Data portal^19^. GYTS is a nationwide cross-sectional survey among high school students to assess behaviors and risk factors associated with tobacco use among adolescents. The survey was conducted among 6680 eligible students in Junior Secondary School (JSS2 and JSS3) and Senior Secondary School (SSS1). The Sierra Leone GYTS uses a standard questionnaire that explores the following topics: tobacco use (smoking and smokeless), cessation, secondhand smoke (SHS), pro- and anti-tobacco media and advertising, access to and availability of tobacco products, and knowledge and attitudes regarding tobacco use. The research design employed in this study has been described in previous studies^14,20^. In summary, a two-stage cluster sampling method was used to collect the nationally representative data. In the first stage, schools were selected based on their enrolment size, and in the second stage, classes were randomly selected in the selected schools. The overall response rate was 79.6%.

### Measures

Our outcome measures include current tobacco use and susceptibility to using tobacco among non-users of tobacco. The current tobacco use was defined as using cigarettes, smoked tobacco products other than cigarettes and smokeless tobacco products in the last 30 days. Current use of cigarettes was determined by respondents’ self-reported response of one or more days to the question, ‘During the past 30 days, on how many days did you smoke cigarettes?’, and the current use of tobacco products other than cigarette was determined by respondents’ saying ‘yes’ to any of the following questions: ‘During the past 30 days, did you use any form of smoked tobacco products other than cigarettes (such as cigar, pipe water pipe and shisha)?’, and ‘During the past 30 days, did you use any form of smokeless tobacco products (e.g. chewing tobacco, snuff, dip)?’.

We accessed susceptibility to using tobacco among non-tobacco users using the algorithm developed by Pierce et al.^21,22^. Non-tobacco users were defined as those who have never used cigarettes, smoked tobacco products other than cigarettes and smokeless tobacco products. Non-tobacco users were identified as those respondents who responded ‘no’ to the any of the following questions: ‘Have you ever tried or experimented with cigarette smoking, even one or two puffs?’, ‘Have you ever tried or experimented with any form of smoked tobacco products other than cigarettes (such as cigar, pipe, shisha or water pipe)?’, and ‘Have you ever tried or experimented with any form of smokeless tobacco products (such as snuff, chewing tobacco)?’. We defined susceptibility to using tobacco by participants’ responses to a 4-point ordinal scale (1=definitely not; 2=probably not; 3=probably yes; and 4=definitely yes), based on the following questions: 1) ‘If one of your best friends offered you a cigarette, would you smoke it?’; and 2) ‘At any time during the next 12 months do you think you will smoke a cigarette?’. Based on the current literature^14,20,23,24^, we developed 13 factors from 27 questions that could likely be linked with current tobacco use and susceptibility to using tobacco among non-tobacco users in the adolescent population in Sierra Leone. These factors included age, sex, grade, money to be spent on an average week, parental, peer smoking, exposure to secondhand smoke (SHS) inside or outside the home, exposure to anti-smoking media messages, exposure to tobacco industry promotions, support for the smoke-free policy, knowledge about harmful effects of smoking and SHS, whether the family discussed the harmful effects of smoking tobacco, and anti-smoking school education. We dichotomized all variables based on previously described methods^24,25^. Details on how the outcome and independent variables were defined and measured in our study are given in the Supplementary file.

### Statistical analysis

We used SPSS v27 to analyze our data. The sample data were weighted to adjust for sampling design effect, non-responses at school, class and student levels, and post-stratification of the sample relative to sex and grade distribution in the population like earlier studies^14,23,26^ using complex sample design. We represent variables using weighted percentages. Given the established gender difference regarding tobacco use, we conducted separate analyses for male and female adolescents. Complex sample logistic regression analyses were used to determine the correlates of current tobacco use and susceptibility to using tobacco among never smokers, for males and females. In regression analyses, all independent variables were considered potential confounders and all were adjusted for, except for the independent variable in question. Adjusted odds ratios (AOR) and respective 95% confidence intervals (CIs) are reported. A p<0.05 was considered significant for all inferential statistics. Hosmer and Lemeshow test was employed to test the goodness-of-fit of our models, and all models were found to have a good fit. The variance inflation factor (VIF) was used to test for multicollinearity among independent variables, and all VIFs were below 1.30.

## RESULTS

### Study population

Table 1 gives the characteristics of the study population. The study included 6680 school-going adolescents. Approximately half (52.1%) were aged 13–15 years and were males (50.7%). Close to a quarter were exposed to parental (24.5%) and peer smoking behavior (24.0%). The majority of males (94.1%) and females (95.7%) favored a smoke-free policy.

**Table 1:** Population characteristics of Sierra Leone Global Youth Tobacco Survey 2017 (N=6680)

*Characteristics*	*Categories*	*Total %*	*Male %*	*Female %*
**Sex**		100	50.7	49.3
**Age** (years)	≤12	5.4	3.7	4.7
	13–15	52.1	50.8	54.9
	≥16	42.5	45.5	40.3
**Class**	JSS2	41.3	41.0	42.1
	JSS3	39.7	40.8	39.5
	SSS1	19.0	18.1	18.4
**Money on average week**	Have money	61.1	60.3	63.4
	No money	38.9	39.7	36.6
**Parental smoking**	Yes	24.5	27.8	21.1
	No	75.5	72.2	78.9
**SHS exposure in your home**	Yes	27.7	30.9	24.6
	No	72.3	69.1	75.4
**SHS exposure outside home**	Yes	58.7	60.9	56.1
	No	41.3	39.1	43.9
**Peer smoking**	Yes	24.0	31.4	15.1
	No	76.0	68.6	84.9
**Exposure to anti-smoking media messages**	Yes	54.7	56.7	52.7
	No	45.3	43.3	47.3
**Support for smoke-free policy**	Yes	94.8	94.1	95.7
	No	5.2	5.9	4.3
**Knowledge about harmful effects of smoking and SHS**	Yes	91.6	90.7	92.8
	No	8.4	9.3	7.2
**Tobacco industry promotion**	Yes	28.0	33.5	22.5
	No	72.0	66.5	77.5
**Family discussed the harmful effects of smoking tobacco**	Yes	58.0	59.3	56.3
	No	42.0	40.7	43.7
**Anti-smoking school education**	Yes	52.0	53.3	50.8
	No	48.0	46.7	49.2

SHS: secondhand smoke.

### Current tobacco use and its determinants

Table 2 gives details of the distribution of current tobacco use and susceptibility to using tobacco among non-users of tobacco in school-going adolescents across the independent variables, stratified by gender. The overall prevalence of current tobacco use among high school adolescents in Sierra Leone was 24.6%, higher in males (27.9%) than in females (18.6%). Interestingly, the highest current prevalence of tobacco use was among adolescents who support a smoke-free policy (96.1%), and this was the case for both sexes (males 95.2% and females 97.0%). Equally, the majority of high school adolescents who said they were knowledgeable about the harmful effects of smoking and SHS, were current users of tobacco products (93.8%) and no significant gender difference was observed (males 95.1% and females 91.7%).

**Table 2:** Distribution of current tobacco use and susceptibility to using tobacco among non-users of tobacco in school-going adolescents, across the independent variables, stratified by gender, Sierra Leone Global Youth Tobacco Survey 2017 (N=6680)

*Characteristics*	*Current user of tobacco products*			*Current user of tobacco products (yes)*		*Susceptibility to using tobacco among non-tobacco users*			*Susceptibility to using tobacco among non-tobacco users (yes)*	
	*Yes*	*No*	*p*	*Male*	*Female*	*Yes*	*No*	*p*	*Male*	*Female*
	*n (%)*	*n (%)*		*n (%)*	*n (%)*	*n (%)*	*n (%)*		*n (%)*	*n (%)*
	1613 (24.6)	5067 (75.4)		800 (27.9)	658 (18.6)	984 (18.2)	4553 (81.8)		418 (18.0)	545 (18.5)
**Age (years)**			0.003					0.083		
≤12	238 (11.8)	157 (3.3)		41 (5.1)	108 (12.3)	24 (2.2)	221 (4.8)		9 (1.8)	11 (1.9)
13–15	719 (49.2)	2554 (53.2)		367 (48.4)	321 (55.4)	477 (50.0)	2296 (53.3)		194 (48.1)	279 (53.0)
≥16	586 (39.0)	2208 (43.5)		358 (46.5)	206 (32.4)	455 (47.8)	1908 (41.9)		202 (50.1)	243 (45.1)
**Grade**			0.663					0.857		
JSS2	590 (41.2)	1836 (41.3)		311 (39.1)	251 (48.8)	367 (41.0)	1658 (41.5)		175 (42.4)	183 (39.6)
JSS3	556 (36.6)	2224 (40.7)		313 (40.3)	212 (33.0)	449 (41.4)	1893 (39.2)		183 (41.1)	262 (43.2)
SSS1	420 (22.2)	947 (18.0)		165 (20.6)	175 (18.2)	155 (17.6)	955 (19.3)		55 (16.5)	96 (17.2)
**Money on average week**			0.319					0.728		
Have money	873 (58.1)	3013 (62.0)		445 (58.7)	381 (61.8)	589 (61.8)	2627 (60.3)		229 (55.7)	357 (69.4)
No money	710 (41.9)	1977 (38.0)		340 (41.3)	271 (38.2)	376 (38.2)	1870 (39.7)		186 (44.3)	175 (30.6)
**Parental smoking**			<0.001					0.353		
Yes	534 (36.0) 1	1037 (20.9)		307 (40.7)	194 (30.8)	231 (24.7)	987 (22.2)		101 (25.7)	129 (25.0)
No	1011 (64.0)	3933 (79.1)		457 (59.3)	441 (69.2)	728 (75.3)	3486 (77.8)		311 (74.3)	399 (75.0)
**SHS exposure in your home**			<0.001					0.504		
Yes	643 (41.8)	1212 (23.2)		372 (48.1)	237 (35.8)	285 (27.9)	1147 (24.9)		111 (25.5)	170 (30.8)
No	924 (58.2)	3797 (76.8)		405 (51.9)	405 (64.2)	683 (72.1)	3360 (75.1)		302 (74.5)	364 (69.2)
**SHS exposure outside home**			0.003					0.765		
Yes	1049 (69.5)	2829 (55.5)		533 (73.3)	396 (61.1)	583 (58.8)	2600 (57.5)		251 (59.4)	327 (60.8)
No	443 (30.5) 2	2149 (44.5)		195 (26.7)	216 (38.9)	371 (41.2)	1841 (42.5)		159 (40.6)	196 (39.2)
**Peer smoking**			<0.001					0.276		
Yes	746 (44.9)	893 (17.3)		408 (50.7)	239 (32.5)	235 (23.7)	980 (21.2)		127 (29.5)	103 (18.3)
No	842 (55.1) 4	4142 (82.7)		378 (49.3)	412 (67.5)	745 (76.3)	3562 (78.8)		290 (70.5)	439 (81.7)
**Exposure to anti-smoking media messages**			0.001					0.131		
Yes	995 (63.4)	2565 (52.0)		498 (64.0)	402 (62.7)	482 (50.1)	2466 (55.6)		250 (62.6)	317 (59.0)
No	550 (36.6)	2415 (48.0)		277 (36.0)	228 (37.3)	483 (49.9)	2016 (44.4)		151 (37.4)	218 (41.0)
**Support for smoke-free policy**			0.139					0.152		
Yes	1333 (96.1)	4557 (94.4)		682 (95.2)	539 (97.0)	874 (92.4)	4097 (95.4)		370 (93.7)	488 (93.1)
No	50 (3.9)	260 (5.6)		31 (4.8)	18 (3.0)	62 (7.6)	196 (4.6)		21 (6.3)	37 (6.9)
**Knowledge about harmful effects of smoking and SHS**			0.033					0.483		
Yes	1411 (93.8)	4493 (90.9)		704 (95.1)	572 (91.7)	895 (92.8)	4025 (91.3)		376 (92.4)	504 (94.6)
No	91 (6.2)	414 (9.1)		37 (4.9)	48 (8.3)	63 (6.3)	359 (8.7)		30 (14.2)	28 (5.4)
**Tobacco industry promotion**			<0.001					0.122		
Yes	512 (38.6)	1234 (24.9)		325 (45.9)	165 (30.8)	288 (30.8)	1119 (25.5)		136 (34.2)	148 (28.4)
No	858 (61.4)	3597 (75.1)		389 (54.1)	358 (69.2)	652 (69.2)	3189 (74.5)		265 (65.8)	373 (71.6)
**Family discussed the harmful effects of smoking tobacco**			0.062					0.378		
Yes	975 (63.9)	2788 (56.1)		492 (64.9)	383 (59.8)	532 (54.5)	2586 (58.4)		244 (59.3)	278 (50.8)
No	530 (36.1)	2135 (43.9)		263 (35.1)	242 (40.2)	422 (45.5)	1827 (41.6)		156 (40.7)	256 (49.2)
**Anti-smoking school education**			0.383					0.027		
Yes	786 (47.7)	2772 (53.3)		391 (49.2)	303 (42.8)	466 (46.4)	2562 (55.3)		210 (48.8)	255 (46.7)
No	723 (52.3)	2246 (46.7)		363 (50.8)	309 (57.2)	511 (53.6)	1896 (44.7)		207 (51.2)	284 (53.3)

SHS: secondhand smoke.

Table 3 summarizes the factors significantly associated with current tobacco use among school-going adolescents in Sierra Leone. Males (AOR=1.50; 95% CI: 1.18–1.91) were more likely to be current tobacco users than females. Adolescents exposed to parental smoking (AOR=1.73; 95% CI: 1.32–2.26), exposed to household secondhand smoke (AOR=1.82; 95% CI: 1.27–2.62), having peers who smoke (AOR=2.24; 95% CI: 1.51–3.31), were more likely to be current users of tobacco. Similar associations between exposure to secondhand smoke at home and current use of tobacco were observed among males (AOR=1.94; 95% CI: 1.31–2.86) and females (AOR=1.64; 95% CI: 1.03– 2.61). A similar relationship between having friends who smoke and being a current tobacco user was observed among males (AOR=2.19; 95% CI: 1.42–3.37) and females (AOR=2.21; 95% CI: 1.40–3.50). Adolescents who were not exposed to anti-smoking media messages were less likely to be current tobacco users (AOR=0.67; 95% CI: 0.52–0.85), although such association was observed among females (AOR=0.51; 95% CI: 0.34–0.77) but not males (AOR=0.80; 95% CI: 0.63–1.03). Interestingly, although no overall significant association was observed between not being knowledgeable about the harmful effects of smoking and SHS and being a current user of tobacco, a gender difference was observed with a more likely association seen among females (AOR=1.79; 95% CI: 1.14–2.81) and less likely association among males (AOR=0.39; 95% CI: 0.17– 0.88). Adolescents not exposed to anti-tobacco education were more likely to be current users of tobacco (AOR=1.81; 95% CI: 1.07–3.05), and such an association was observed among females (AOR=2.14; 95% CI: 1.15–3.97) but not males (AOR=1.63; 95% CI: 0.96–2.77).

**Table 3:** Determinants of current tobacco use and susceptibility to using tobacco among non-users of tobacco in school-going adolescents, stratified by gender, Sierra Leone Global Youth Tobacco Survey 2017 (N=6680)

*Determinants*	*Current use of tobacco*			*Susceptibility to using tobacco among non-tobacco users*		
	*Total*	*Male*	*Female*	*Total*	*Male*	*Female*
	*AOR (95% CI)*	*AOR (95% CI)*	*AOR (95% CI)*	*AOR (95% CI)*	*AOR (95% CI)*	*AOR (95% CI)*
**Sex**						
Male	1.50 (1.18–1.91)*			0.96 (0.75–1.23)		
Female (Ref.)	1			1		
**Age** (years)						
≤12 (Ref.)	1	1	1	1	1	1
13–15	0.59 (0.39–0.90)	0.50 (0.30–0.84)	0.67 (0.30–1.55)	1.46 (0.82–2.59)	1.61 (0.71–3.64)	1.27 (0.62–2.63)
≥16	0.59 (0.37–0.94)	0.54 (0.32–0.94)	0.55 (0 .22–1.36)	1.64 (0.89–3.01)	1.97 (0.84–4.61)	1.36 (0.63–2.89)
**Grade**						
JSS2 (Ref.)	1	1	1	1	1	1
JSS3	0.98 (0.61–1.56)	1.16 (0.68–1.99)	0.80 (0.48–1.34)	1.13 (0.72–1.77)	0.94 (0.60–1.47)	1.33 (0.72–2.44)
SSS1	1.05 (0.59–1.88)	1.46 (0.70–3.04)	0.58 (0.37–0.93)	0.84 (0.45–1.54)	0.67 (0.35–1.29)	1.02 (0.44–2.32)
**Money to be spent on average week**						
Have money	0.92 (0.73–1.16)	0.80 (0.58–1.11)	1.12 (0.74–1.70)	1.10 (0.79–1.52)	0.83 (0.61–1.14)	1.45 (0.96–2.19)
No money (Ref.)	1	1	1	1	1	1
**Parental smoking**						
Yes	1.73 (1.32–2.26)*	1.64 (1.25–2.17)*	2.00 (1.35–2.95)	0.87 (0.62–1.21)	0.88 (0.55–1.40)	0.85 (0.60–1.21)
No (Ref.)	1	1	1	1	1	1
**SHS exposure in your home**						
Yes	1.82 (1.27–2.62)**	1.94 (1.31–2.86)**	1.64 (1.03–2.61)*	1.12 (0.69–1.84)	0.80 (0.46–1.41)	1.57 (0.92–2.70)
No (Ref.)	1	1	1	1	1	1
**SHS exposure outside home**						
Yes	1.00 (0.62–1.61)	1.21 (0.72–2.01)	0.80 (0.47–1.37)	1.08 (0.77–1.53)	0.96 (0.59–1.56)	1.25 (0.86–1.82)
No (Ref.)	1	1	1	1	1	1
**Peer smoking**						
Yes	2.24 (1.51–3.31)*	2.19 (1.42–3.37)*	2.21 (1.40–3.50)*	1.10 (0.79–1.54)	1.01 (0.71–1.45)	1.46 (0.87–2.43)
No (Ref.)	1	1	1	1	1	1
**Exposure to antismoking media messages**						
Yes (Ref.)	1	1	1	1	1	1
No	0.67 (0.52–0.85)*	0.80 (0.63–1.03)	0.51 (0.34–0.77)*	1.19 (0.88–1.60)	1.15 (0.79–1.63)	1.26 (0.89–1.78)
**Support for smoke-free policy**						
Yes (Ref.)	1	1	1	1	1	1
No	0.69 (0.41–1.15)	0.82 (0.43–1.57)	0.43 (0.21– 0.90)	1.15 (0.66–2.01)	0.70 (0.33–1.51)	1.94 (1.10–3.45)*
**Knowledge about harmful effects of smoking and SHS**						
Yes (Ref.)	1	1	1	1	1	1
No	0.70 (0.41–1.18)	0.39 (0.17–0.88)	1.79 (1.14–2.81)*	0.55 (0.33–0.92)*	0.57 (0.27–1.18)	0.46 (0.24–0.89)*
**Tobacco industry promotion**						
Yes	1.31 (0.98–1.75)	1.28 (0.94–1.75)	1.26 (0.75–2.10)	1.50 (1.06–2.10)*	1.28 (0.84–1.96)	1.88 (1.20–2.94)*
No (Ref.)	1	1	1	1	1	1
F**amily discussed the harmful effects of smoking tobacco**						
Yes (Ref.)	1	1	1	1	1	1
No	0.95 (0.63–1.44)	0.91 (0.58–1.42)	1.00 (0.61–1.63)	1.01 (0.71–1.43)	0.86 (0.58–1.28)	1.12 (0.77–1.62)
**Antismoking school education**						
Yes (Ref.)	1	1	1	1	1	1
No	1.81 (1.07–3.05)	1.63 (0.96–2.77)	2.14 (1.15–3.97)*	1.39 (1.05–1.86)*	1.45 (1.05–2.00)*	1.39 (0.99–1.96)

* p<0.05.

** p<0.001.

SHS: secondhand smoke. AOR: adjusted odds ratio. In the regression analyses all independent variables were considered as potential confounders and all were adjusted for, except for the independent variable in question.

### Adolescent susceptibility to smoking tobacco and its determinants

The overall tobacco non-users susceptibility to using tobacco among adolescents in Sierra Leone was 18.2% (males 18.0%, females 18.5%) (Table 2). The highest prevalence of susceptibility to using tobacco among non-users of tobacco was among those who were knowledgeable about the harmful effects of smoking and secondhand smoke (92.8%), followed by those who showed support for smoke-free policy (92.4%), and those who had peers that do not smoke (76.3%). A similar pattern was observed among males and females.

Table 3 shows the factors that were associated with susceptibility to using tobacco among non-users of tobacco. Two factors were significant determinants of susceptibility to using tobacco among non-tobacco users. Adolescents who were exposed to tobacco promotion (AOR=1.50; 95% CI: 1.06–2.10) and those who were not exposed to anti-smoking education in school (AOR=1.39; 95% CI: 1.05–1.86) were more likely than their reference groups to be susceptible to using tobacco. Among males, those not exposed to anti-smoking education in school were more likely to be susceptible to using tobacco (AOR=1.45; 95% CI: 1.05–2.00) compared to those who were exposed to anti-smoking education in school. Among females, those exposed to tobacco industry promotion (AOR=1.88; 95% CI: 1.20–2.94) and those not in support for smoke-free policy (AOR=1.94; 95% CI: 1.10–3.45) were more likely to be susceptible to tobacco use among non-users compared to their reference groups. On the other hand, adolescents who were not knowledgeable about the harmful effects of smoking and secondhand smoke were less likely to be susceptible to tobacco use among non-users (AOR=0.55; 95% CI: 0.33–0.92) compared to those who were knowledgeable about the harmful effects of smoking and secondhand smoke. A similar association between not being knowledgeable about the harmful effects of smoking and secondhand smoke and susceptibility to tobacco use among non-users was observed among females (AOR=0.46; 95% CI: 0.24–0.89) but not males (AOR=0.57; 95% CI: 0.27–1.18).

## DISCUSSION

Tobacco use is a significant risk factor for an increasing burden of non-communicable diseases in Sub-Saharan Africa. Given that non-communicable diseases are on the rise among the adult population in Sierra Leone, most of these adults start to smoke when they are teenagers. Such tobacco use pattern warrants exploring adolescents’ current tobacco use and susceptibility to using tobacco among non-users to inform policy, public health interventions and advocacy. Our study indicates that one in four high school adolescents aged 11–17 years in Sierra Leone was a current tobacco user 30 days before this survey. The current prevalence of current tobacco use among males and females is higher than those reported in similar studies in Ghana^26^, Gambia^27^, and Sudan^28^, but lower than those in Madagascar^23^ and the Republic of Congo^29^. Differences in prevalence maybe attributed to variations in how tobacco use was measured, and the age group considered. As reported among Sierra Leone adults^30^, the relatively high use of tobacco products among school-going adolescents is a public health concern and indicates the need for youth-centered tobacco policies and interventions. As reported in other studies^26,28^, we also observed a gender difference regarding tobacco use behavior among school-going adolescents in Sierra Leone. The difference in tobacco use prevalence between males and females warrants the inclusion of gender considerations when designing and implementing tobacco policies targeting youth in Sierra Leone. Interventions such as age verification and a ban on tobacco products to minors could serve as deterrents to restrict youth access to and demand for tobacco products.

In line with current literature^3^, results from our regression analysis indicated that males were more likely than females to be current users of tobacco, which may be attributed to a combination of social, cultural, and behavioral factors^31^. In many societies, smoking among females is considered undesirable, and they are more likely to believe that society disapproves of smoking^32^. Also, males have the false perception that smoking makes them more attractive among their peers^32^. Adolescents whose parents were smokers and were exposed to secondhand smoke in the home, were more likely to be current tobacco users, and this was observed for both sexes. This suggests that family relations are a strong influencing factor associated with tobacco use behavior. This is in line with studies from Ghana, Sudan, and South Africa, where familial relationships were found to be a significant factor associated with youth tobacco use^26,28,33^. Consistent with other studies^23,26^, peer smoking behavior was a significant predictor of current users of tobacco in our study, and such an association was observed in both sexes. Our findings suggest that interventions need to be developed to target the familial relationships between adolescents and their peers. Family and peer education will be useful interventions to help curb the family and peer influence on youth smoking behavior in Sierra Leone. Also, our findings suggest the need for community-based tobacco prevention interventions to limit tobacco use among young people. Community-based tobacco prevention interventions are effective in limiting the uptake of tobacco in young people^34^. We did not observe any significant association between youth exposure to tobacco industry promotion and current tobacco use, as reported elsewhere^26^. This further suggests that tobacco promotion and advertisement might not be significantly influencing school-going adolescent tobacco use behavior. Notwithstanding that, it is essential that the government implement a ban on tobacco promotion and advertisement along with other MPOWER strategies to help protect citizens from the harmful effects of tobacco use^9^.

Also, gender disparity was noted regarding exposure to anti-smoking education in schools in our study. We found that non-exposure to anti-smoking education in school was a significant determinant of current tobacco use status among females but not males, which implies the need for an education policy that makes teaching about the harmful effects of tobacco use mandatory in schools.

Our study also showed that close to one in five non-users of tobacco in Sierra Leone aged 11–17 years was susceptible to using tobacco products, although there was no significant gender difference. Our prevalence is higher than the global prevalence of 12.5%^14^ and the African prevalence of 12.2%^3^. Such a result suggests the need to develop interventions that prevent non-tobacco users from using tobacco and eventually becoming regular users by targeting them at the pre-experimental stage. In line with previous studies^3,14^, tobacco industry promotion is a significant factor influencing susceptibility to using tobacco products among non-users in our study. The strong influence of the tobacco industry on the intention to use tobacco among adolescent has been recognized by the World Health Organization in 2012 World No Tobacco Day, themed ‘Tobacco industry interference’, which exposes and advocates a ban on tobacco industry attempts to undermine global tobacco policy initiatives as enshrined in the WHO FCTC treaties^9^. To reduce the tobacco industry’s influence on the intention to use tobacco among non-tobacco users, it is important that the Sierra Leone government take steps to impose a total ban on tobacco advertisement and promotion in line with the WHO Framework Convention on Tobacco Control (FCTC)^9^. Also, non-exposure to anti-smoking education in school was a significant factor influencing susceptibility to use tobacco products among non-users of tobacco, which is consistent with a study in African countries in which students exposed to educational programs on the health effects of smoking were less likely to be susceptible to smoking^3^. Our findings suggests that teaching about the adverse effects of tobacco use should be part of the national school curriculum in Sierra Leone. Also, female adolescents who are not in favor of a smoke-free policy or ban on the use on tobacco in public places, sale of tobacco products to minors and tobacco advertising, were likely to be susceptible to using tobacco products among non-users in our study. This maybe because of the significant strong influence of tobacco industry promotion observed in this study, which would influence their perceptions regarding the use of tobacco products.

### Limitations

This study has some limitations. First, our results are not representative of all adolescents, given that only school-going adolescents were targeted and those present on the day this survey was administered. Second, our study employed a cross-sectional study design, which limits any causal relationship to be inferred. Third, we used 27 questions to construct 14 predictors based on the available scholarship on tobacco use among school-going adolescents. Country-specific measures such as national tobacco control programs or policies were not included. Future studies on this topic in Sierra Leone should include country-specific variables that might potentially influence youth tobacco use. Fourth, our study is also prone to recall bias as the data collected was based on self-report. Finally, we reported our findings as current even though the data were collected in 2017, though this study provides the latest picture regarding adolescent tobacco use. However, future GYTS surveys are needed to follow the trends in adolescent tobacco use and understand the impact of national tobacco policies and interventions on adolescent tobacco use patterns. Notwithstanding these limitations, our study is the first to estimate the prevalence and factors associated with current tobacco use and susceptibility to using tobacco products among non-users among school-going adolescents aged 11–17 years, using national representative data.

## CONCLUSIONS

Our study suggests that one in four school-going adolescents is a current tobacco user. Also, close to one in five non-users of tobacco aged 11–17 years was susceptible to using tobacco products. There is gender disparity among current tobacco users, but not among non-users of tobacco, with regard to susceptibility to using tobacco products. Irrespective of gender, exposure to secondhand smoke at home and peer smoking were significant determinants of current tobacco use. Exposure to tobacco industry promotion and non-exposure to anti-smoking education in school were significant factors associated with susceptibility to using tobacco products among non-users in our study. Given the high use of tobacco products among Sierra Leone adults, our findings have provided evidence that showcases the need for the government and other stakeholders to implement policies and interventions to prevent tobacco use among adolescents in Sierra Leone, with the aim of protecting them from becoming tobacco users in their adult life.

## Data Availability

The data supporting this research are available from the following sources: https://extranet.who.int/ncdsmicrodata/index.php/catalog/783/ study-description
