# Supplementary material for: Current tobacco use and susceptibility to using tobacco among non-users of tobacco: A cross-sectional study among school-going adolescents in Sierra Leone
Source: Tob Induc Dis. 2023 Jan 30;21:16. doi: 10.18332/tid/157091 (PMC9885443; doi:10.18332/tid/157091)
Supplement: Supplementary file 1 [file TID-21-16-s1.pdf]

Supplementary material

Additional file 1:

Table 1: Study measures, survey items with responses, Sierra Leone Global Youth Tobacco Survey (GYTS)

Study measure	GYTS survey items	GYTS item responses	Dichotomized measure	
GYTS question used to obtain never-non-tobacco users' status	Have you ever tried or experimented with cigarette smoking, even one or two puffs?",	Yes<br>No	Yes- Ever smoker<br>No-Never smoker	No- no for all three dichotomized items<br><br>Yes - for all three dichotomized items
	Have you ever tried or experimented with any form of smoked tobacco products other than cigarettes	Yes<br>No	Yes- Ever smoker<br>No-Never smoker	
	Have you ever tried or experimented with any form of smokeless tobacco products (such as snuff, chewing tobacco)?"	Yes<br>No	Yes- Ever use smokeless tobacco<br>No - Never used smokeless tobacco	
Dependent variable Tobacco use susceptibility	If one of your friends offered you a cigarette, would you smoke it?	Definitely not<br>Probably not<br>Probably yes<br>Definitely yes	No "Definitely not" for all three items<br><br>Yes any other responses for any of the three item	
	At anytime during the next 12 months do you think you will use any form of tobacco?	Definitely not<br>Probably not<br>Probably yes<br>Definitely yes	No "Definitely not" for all three items<br><br>Yes any other responses for any of the three item	
Dependent variable: current tobacco use	During the past 30 days, on how many days did you smoke	0 days<br>1 or 2 days<br>3 to 5 days<br>6 to 9 days<br>10 to 19 days	No- 0 days<br>Yes- 1 or more days	No- no for all three

	cigarettes?	20 to 29 days<br>All 30 days		dichotomized items
	During the past 30 days, did you use any form of smoked tobacco products other than cigarettes (such as cigar, pipe water pipe and shisha)?	Yes<br>No	Yes<br>No	Yes - for all three dichotomized items
	During the past 30 days, did you use any form of smokeless tobacco products (e.g. chewing tobacco, snuff, dip)?	Yes<br>No	Yes<br>No	
**Independent variables**				
Age	How old are you?	11 years old or younger<br>12 years old<br>13 years old<br>14 years old<br>15 years old<br>16 years old<br>17 years old or older	≤13 years old<br><br>≥14years old	
Sex	What is your sex?	Male<br>female	Male<br>female	
Grade / form	In what grade/form are you?	JSS2<br>JSS3<br>SSS1	JSS2<br>JSS3<br>SSS1	
Money to be spent on average week	During an average week, how much money do you have that you can spend on yourself, however you want?	I usually don't have any spending money<br>Less than Le 20,000<br>Le 20,000 - Le 40,000<br>Le 41,000 - Le 60,000<br>Le 61,000 - Le 80,000<br>Le 81,000 - Le 100,000<br>Le 101,000 -Le	No Money- I usually don't have any spending money<br><br>Have money- any other responses for any of the remaining six items	

		500,000		
Parental smoking	How often do you see your father (stepfather or mother's partner) smoking in your home?	Don't have/Don't see this person<br>About every day<br>Sometimes<br>Never	No- Don't have/Don't see this person and Never<br><br>Yes- About every day and Sometimes	No- no for both dichotomized items
	How often do you see your mother (stepmother or father's partner) smoking in your home	Don't have/Don't see this person<br>About every day<br>Sometimes<br>Never	No- Don't have/Don't see this person and Never<br><br>Yes- About every day and Sometimes	Yes - for both dichotomized items
Peer smoking	Do any of your closest friends smoke tobacco?	None of them<br>Some of them<br>Most of them<br>All of them	No- None of them<br>Yes- Some of them<br>Most of them<br>All of them	
SHSb exposure in your home	During the past 7 days, on how many days has anyone smoked inside your home, in your presence?	0 days<br>1 to 2 days<br>3 to 4 days<br>5 to 6 days<br>7 days	No- 0 days<br>Yes-1 to 2 days<br>3 to 4 days<br>5 to 6 days<br>7 days	
SHSb exposure outside home	During the past 7 days, on how many days has anyone smoked in your presence, inside any enclosed public place, other than your home (such as office, school, shops, restaurants, cinemas, night club)?	0 days<br>1 to 2 days<br>3 to 4 days<br>5 to 6 days<br>7 days	No- 0 days<br>Yes-1 to 2 days<br>3 to 4 days<br>5 to 6 days<br>7 days	No- no for all three dichotomized items<br><br>Yes - for all three dichotomized items
	During the past 7 days, on how many days has	I did not use public transportation during the past 7 days	No-" I did not use public transportation during the past 7	

	anyone smoked in your presence, inside any public transportation vehicles, such as trains, buses, or taxicabs?	I used public transportation, but no one smoked in my presence<br>1 to 2 days<br>3 to 4 days<br>5 to 6 days<br>7 days	days" and "I used public transportation, but no one smoked in my presence"<br><br>Yes-1 to 2 days<br>3 to 4 days<br>5 to 6 days<br>7 days	
	During the past 7 days, on how many days has anyone smoked in your presence, at any outdoor public place (such as playgrounds, sidewalks, entrances to buildings, parks, beaches, vehicle)?	0 days<br>1 to 2 days<br>3 to 4 days<br>5 to 6 days<br>7 days	No- 0 days<br>Yes-1 to 2 days<br>3 to 4 days<br>5 to 6 days<br>7 days	
Exposure to antismoking media messages	During the past 30 days, how many antismoking media messages (e.g., television, radio, billboards, posters, newspapers, magazines, movies) have you seen?	Yes<br>No	Yes<br>No	No- no for both dichotomized items<br><br>Yes - for both dichotomized items
Support for smoke-free policy	Are you in favour of banning smoking in public places (such as in restaurants, in buses, streetcars, and trains, in schools, on	No<br>Yes	No<br>Yes	No- no for all three dichotomized items

	playgrounds, in gyms and sports arenas, in discos)?			Yes - for all three dichotomized items
	Do you think the sale of tobacco products to minors should be banned?	No<br>Yes	No<br>Yes	
	Do you think tobacco advertising should be banned?	No<br>Yes	No<br>Yes	
Knowledge about harmful effects of smoking and SHS	Do you think cigarette smoking is harmful to your health?	Definitely not<br>Probably not<br>Probably yes<br>Definitely yes	No "Definitely not" for both items<br><br>Yes - any other responses for either item	No- no for both dichotomized items<br><br>Yes - for both dichotomized items
	Do you think the smoke from other people's cigarettes is harmful to you?	Definitely not<br>Probably not<br>Probably yes<br>Definitely yes	No "Definitely not" for both items<br><br>Yes - any other responses for either item	
Tobacco industry promotion	Do you have something (t-shirt, pen, backpack, etc.) with a cigarette brand logo on it?	No<br>Yes	No<br>Yes	No- no for both dichotomized items<br><br>Yes - for both dichotomized items
	Has a (cigarette representative) ever offered you a free cigarette?	No<br>Yes	No<br>Yes	
Family discussed the harmful effects of smoking tobacco	Has anyone in your family discussed the harmful effects of smoking tobacco	No<br>Yes	No<br>Yes	

	with you?			
Antismoking school education	During the past 12 months, were you taught in any of your classes about the dangers of tobacco use?	No<br>I don't know<br>Yes	No- No<br>I don't know<br><br>Yes- Yes	

© 2023 James P.B. et al.
